# University of Maryland Dental Department

**Published:** 1889-07

**Authors:** 


### Editorial, Etc.

University of Maryland Dental Department.—*Addition to Dental Building.*—Owing to the steady increase each year in the number of dental students, the Faculty have again found it necessary to add an addition to the Dental Building, which is now in process of construction, and will be completed during the month of August.

This second addition is 44 by 31 feet, giving a Dental Infirmary nearly *one hundred feet long*, and lighted by *forty-three* large windows. The Laboratory will be increased some 31 feet in length over the present size, with a width in the new addition of forty-four feet. A Museum Hall will also be constructed on the second story thirty feet long by fifteen feet wide, the present one on the first floor being converted into an Impression Room, thereby affording an extra Waiting Room for patients for Laboratory Work in the present Impression Room. This addition will give the Dental Department of the University of Maryland the largest Dental Infirmary and Dental Laboratory in existence, and affords the best lighted halls employed for such purposes. Owing to the location of this Dental Building, the light is onobstructed, so that dental chairs and laboratory desks placed in any part of the Infirmary and Laboratory can be used at all times during the day, and, by means of artificial light, even at night when necessity requires. An unusually large number of students have already matriculated for the coming session of 1889 90, and the prospects for a very large class are encouraging.

